# Exploring the Influence of Cation and Halide Substitution in the Structure and Optical Properties of CH_3_NH_3_NiCl_3_ Perovskite

**DOI:** 10.3390/molecules29092141

**Published:** 2024-05-05

**Authors:** Natalí Navarro, Ronald Nelson, Karem Gallardo, Rodrigo Castillo

**Affiliations:** 1 Departamento de Química, Facultad de Ciencias, Universidad Católica del Norte, Avda. Angamos 0610, Antofagasta 1270709, Chile; natali.navarro@ce.ucn.cl (N.N.); rnelson@ucn.cl (R.N.); 2 Instituto de Ciencias Aplicadas, Facultad de Ingeniería, Universidad Autónoma de Chile, Av. El Llano Subercaseaux 2801, Santiago 8910060, Chile; karem.gallardo.4@gmail.com; 3 Departamento de Química Inorgánica, Facultad de Química y de Farmacia, Pontificia Universidad Católica de Chile, Vicuña Mackenna 4860, Santiago 7820436, Chile

**Keywords:** hybrid perovskites, halogen substitution, optical absorption

## Abstract

This manuscript details a comprehensive investigation into the synthesis, structural characterization, thermal stability, and optical properties of nickel-containing hybrid perovskites, namely CH_3_NH_3_NiCl_3_, CsNiCl_3_, and CH_3_NH_3_NiBrCl_2_. The focal point of this study is to unravel the intricate crystal structures, thermal behaviors, and optical characteristics of these materials, thereby elucidating their potential application in energy conversion and storage technologies. X-ray powder diffraction measurements confirm that CH_3_NH_3_NiCl_3_ adopts a crystal structure within the *Cmcm* space group, while CsNiCl_3_ is organized in the *P6_3_/mmc* space group, as reported previously. Such structural diversity underscores the complex nature of these perovskites and their potential for tailored applications. Thermal analysis further reveals the stability of CH_3_NH_3_NiCl_3_ and CH_3_NH_3_NiBrCl_2_, which begin to decompose at 260 °C and 295 °C, respectively. The optical absorption properties of these perovskites studied by UV-VIS-NIR spectroscopy revealed the bands characteristic of Ni^2+^ ions in an octahedral environment. Notably, these absorption bands exhibit subtle shifts upon bromide substitution, suggesting that optical properties can be finely tuned through halide modification. Such tunability is paramount for the design and development of materials with specific optical requirements. By offering a detailed examination of these properties, the study lays the groundwork for future advancements in material science, particularly in the development of innovative materials for sustainable energy technologies.

## 1. Introduction

Perovskite-structured materials have garnered significant attention due to their diverse range of properties and potential applications. This interest arises from their inherent versatility, which is attributed to their general formula, ABX_3_. Here, A represents a large cation carrying a +1 charge, B denotes a small cation with a +2 charge, and X signifies an anion with a −1 charge. Hybrid organic-inorganic halide perovskites, exemplified by CH_3_NH_3_PbI_3_ and its derivatives, are prominent examples in contemporary research. These materials have been extensively studied as light-harvesting components in third-generation solar cells, demonstrating a remarkable increase in photoconversion efficiency from a modest 5% to over 30% in just a few years [1]. However, despite their impressive performance in solar energy applications, methylammonium (MA = CH_3_NH_3_^+^) lead halide perovskites face two critical challenges: material instability and the toxicity associated with lead, a hazardous heavy metal. In response, researchers have sought alternative materials to replace lead, yet the photoconversion efficiency of these substitutes has not yet matched that of lead-containing compounds.

Nevertheless, ongoing research has unveiled other potential applications for hybrid perovskites, including use in detectors, photocatalysts, fuel cells, and more. The specific application of hybrid perovskite materials often depends on the nature of the B^2+^ cation, allowing for the design of target-oriented compounds tailored for specific purposes. Transition metal-based hybrid perovskites and perovskite-like structures have been explored for various applications, including photovoltaics [2,3,4,5,6] and beyond, such as detectors [7], photocatalysts [8], fuel cells [9,10], light-emitting-diodes [11,12,13], among others [14,15,16,17]. Some transition metal-based perovskites used in fields different from photovoltaics are: (MA)_2_FeCl_4_, which experiences bipolar resistive switching behavior [18]; (MA)_2_CoBr_4_ shows promising electrochemical conversion of water to oxygen capacity [19]; (MA)_2_MnCl_4_ is reported to exhibit red photoluminescence [20]; (MA)_2_FeCuI_4_Cl_2_ and (MA)_2_InCuI_6_ are reported to be functional for ultraviolet photodetector [21]; (MA)_2_HgCl_4_ is reported to be a self-driven ultraviolet photodetector and photoconductor [22]; CsCu_2_I_3_ emits white light [23]; and (MA)NiCl_3_ was tested as active material for lithium-ion batteries [24], to cite a few.

Despite the extensive exploration of transition metal-based perovskites for various applications, there is a noticeable gap in both basics and applied research concerning nickel-containing perovskites, particularly those involving methylammonium or cesium nickel halide compositions. Noteworthy is the work by Poeppelmeier et al., who first synthesized the CsNiX_3_ (X = Cl, Br, I) family by hydrothermal method [25]. Prior to this, the single phases were reportedly obtained solely through the prolonged melting of the nickel halide with cesium halide. Additionally, the contributions of Ramirez et al., who fabricated solar cells using CH_3_NH_3_NiCl_3_ [26] and demonstrated improved efficiency through halide substitution, as well as their use as active material in Li-ion batteries [24], highlighting the potential to avoid cobalt, deserve mention. However, the characterization of these compounds has not been sufficiently comprehensive to fully understand their applications.

Therefore, this paper aims to fill these gaps by focusing on the synthesis, structural elucidation, thermal analysis, optical properties, and vibrational spectroscopy of nickel perovskites, specifically CH_3_NH_3_NiCl_3_, CsNiCl_3_, and CH_3_NH_3_NiBrCl_2_. Through a systematic exploration of property variations across different compositions, this study provides a comprehensive understanding of these materials, paving the way for their potential applications in various energy fields.

## 2. Results and Discussion

### 2.1. Structural Characterization

**Figure 1:**
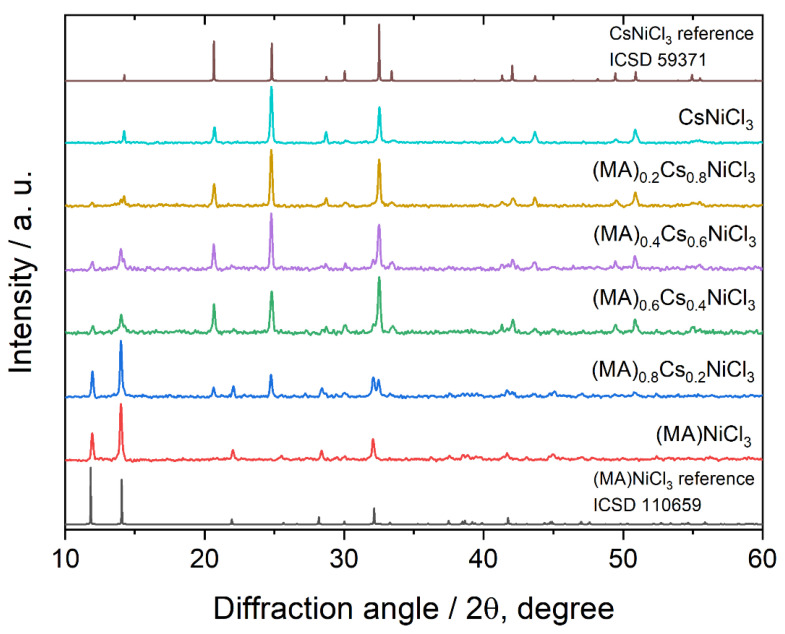
X-ray powder diffraction patterns of samples of mixed samples of composition (MA)_1−x_Cs_x_NiCl_3_, and references patterns for CsNiCl_3_ and (MA)NiCl_3_.

Samples of (CH_3_NH_3_)_1−x_Cs_x_NiCl_3_ (x = 0.0, 0.2, 0.4, 0.6, 0.8. 1.0) all exhibit orange color and are sensitive to air and moisture. The degree of moisture sensitivity was found to be proportional to the amount of CH_3_NH_3_^+^ in the compound, with CsNiCl_3_ being the most stable compound in the series. Due to their sensitivity, all sample handling was conducted under an argon atmosphere. X-ray powder diffraction analysis, Figure 1, revealed that the sample with composition CH_3_NH_3_NiCl_3_ (x = 0.0) crystallizes in the *Cmcm* space group (No. 63) [27], while the sample with composition CsNiCl_3_ (x = 1.0) crystallizes in the *P6_3_/mmc* space group (No. 194) [28]. Since these two compounds adopt different crystal structures, a full range of solid solutions is not expected, which is consistent with the powder diffraction patterns of samples with mixed composition.

**Figure 2:**
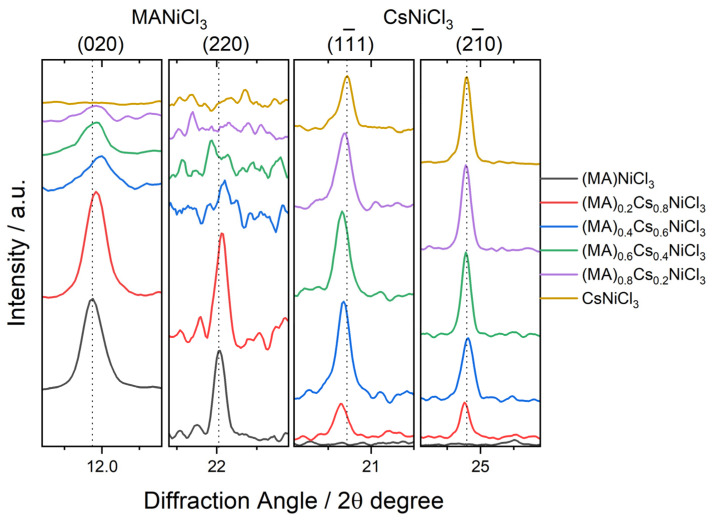
Selected diffraction peaks corresponding to the MANiCl_3_ and CsNiCl_3_ phases, demonstrating discrete shifts with increasing concentrations of Cs^+^ and MA^+^ ions, respectively.

Closer inspection of the diffraction peaks revealed small displacements, as shown in Figure 2. The selected diffraction peaks were used to detect small shifts in the diffraction angles. The lattice planes (1$\bar{1}$1) and (2$\bar{1}$0) of the hexagonal phase CsNiCl_3_ show a discrete shift towards lower angles as the amount of MA^+^ increases, while the lattice planes (020) and (110) of the orthorhombic (MA)NiCl_3_ display a modest shift towards higher angles as the concentration of Cs^+^ increases. Based on these findings, the lattice parameters for samples of single and mixed phases were refined using the LeBail method and are summarized in Table 1.

In order to evaluate the effect of the halogen atom, a sample with composition CH_3_NH_3_NiBrCl_2_ was prepared using the same method as described for the previous compounds, but starting from methylammonium bromide and nickel chloride, following the equation:

CH_3_NH_3_Br + NiCl_2_ → CH_3_NH_3_NiBrCl_2_ (1)

**Figure 3:**
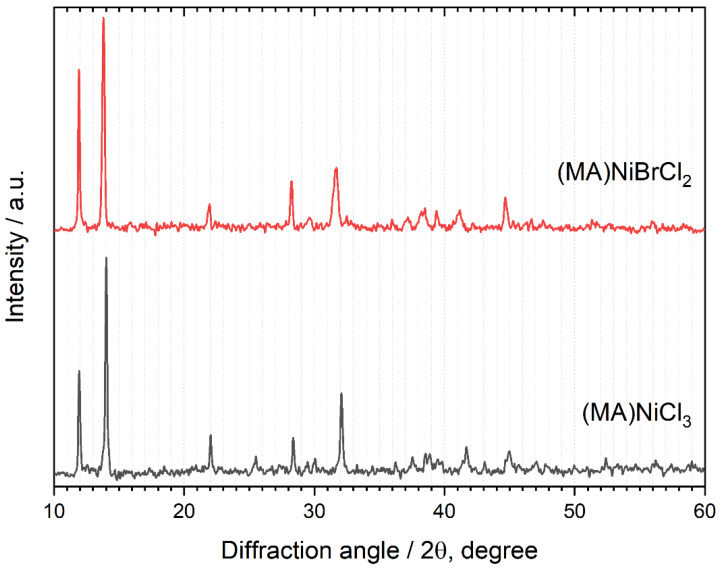
X-ray powder diffraction patterns of samples (MA)NiCl_3_ and (MA)NiBrCl_2_.

The resulting diffraction pattern, shown in Figure 3, confirms the formation of the compound, which adopts the same crystal structure as CH_3_NH_3_NiCl_3_. Lattice parameters derived from Le Bail refinement of the (MA)NiBrCl_2_ pattern yield the values *a* = 7.100 (12), *b* = 14.80 (3), *c* = 6.035 (13), and *V* = 634 (2), representing a 3% increase in the unit cell volume compared to (MA)NiCl_3_ (Table 1). This increase is attributed to the larger radii of the bromide ion.

### 2.2. Thermal Behavior

**Figure 4:**
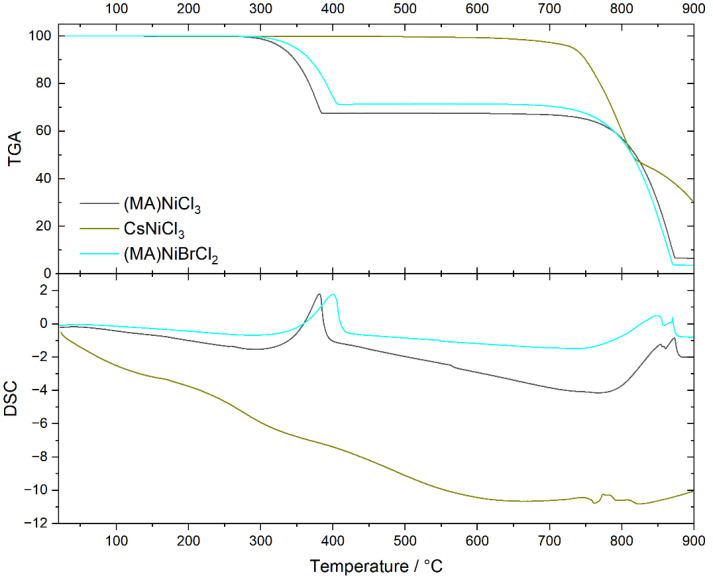
Thermogravimetric (**top**) and differential scanning calorimetry (**bottom**) analysis of samples CsNiCl_3_, (MA)NiCl_3_, and (MA)NiBrCl_2_.

Thermogravimetric analysis of the samples CsNiCl_3_, (MA)NiCl_3_, and (MA)NiBrCl_2_ (Figure 4, top) indicates that CH_3_NH_3_NiCl_3_ begins to decompose at 260 °C, losing 33.92% of its mass, after which it melts at 670 °C, while CsNiCl_3_ remains stable up to about 750 °C when it melts. On the other hand, CH_3_NH_3_NiBrCl_2_ starts to decompose at 295 °C, losing 26.89% of its mass, and then melts at 680 °C. All the thermal effects are associated with DSC endothermic signals, as indicated in the bottom of Figure 4. The melting point of CsNiCl_3_ is in good agreement with the value determined by Boston et al. [29].

The thermal behavior of sample CH_3_NH_3_NiCl_3_ shows a mass loss of 33.92%. which is consistent with the expected mass of CH_3_NH_3_Cl, 34.2%, and the onset temperature of 260 °C is slightly above its boiling point, 230 °C. However, in the case of CH_3_NH_3_NiBrCl_2_, the mass loss of 26.89% corresponds very closely to the molar mass of CH_3_NH_3_Cl (27.95%), suggesting that the resulting product is NiBrCl. According to Equation (1), the decomposition of this hybrid perovskite is not a reversible reaction but follows the reaction:

CH_3_NH_3_NiBrCl_2_ → CH_3_NH_3_Cl + NiBrCl (2)

**Figure 5:**
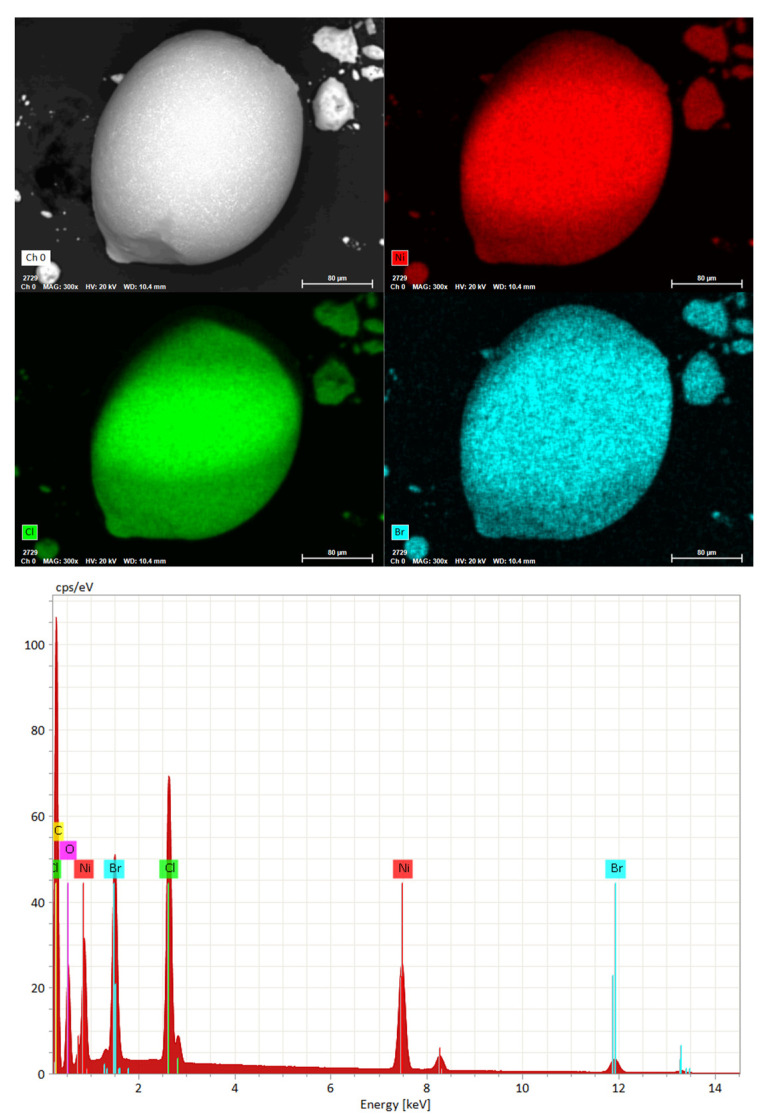
SEM micrograph with EDS mapping of the product after thermal analysis (**top**) and the EDX spectra (**bottom**). Red, green, and cyan correspond to the elements Ni, Cl, and Br, respectively.

This unexpected finding is further supported by chemical analysis performed on the product after the TGA-DSC analysis. Chemical analysis by ICP-OES as well as SEM-EDX measurements, Figure 5, confirm the presence of Ni, Cl, and Br in the products, with a composition closely matching the NiBrCl formula (Br:Cl ≃ 1:1), thereby confirming the proposed decomposition mechanism of this compound.

**Figure 6:**
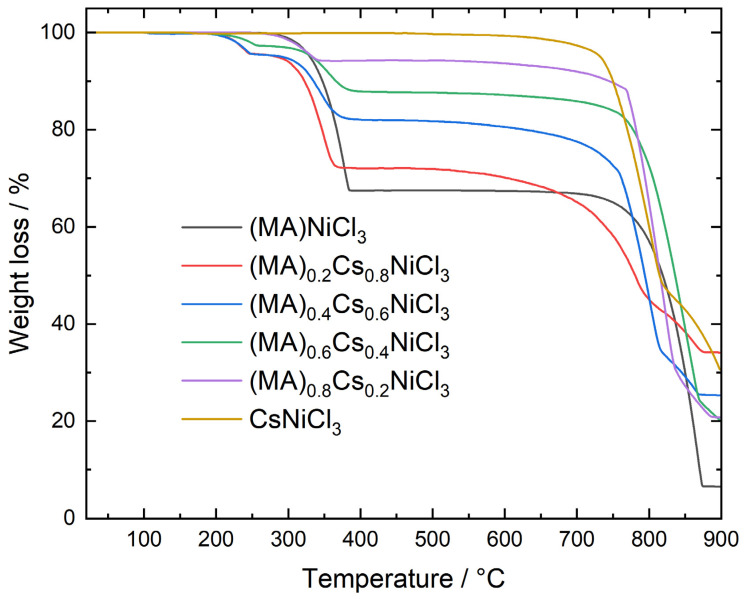
Thermogravimetric behavior of samples (MA)_1−x_CsNiCl_3_ (x = 0.0; 0.2; 0.4; 0.6; 0.8; and 1.0).

The thermal behavior of samples with mixed compositions, as shown in Figure 6, can be understood as follows: (MA)NiCl_3_ decomposes at 260 °C, releasing CH_3_NH_3_Cl, while CsNiCl_3_ melts at 760 °C. Samples with mixed compositions demonstrate mass losses corresponding to the evaporation of CH_3_NH_3_Cl, proportional to their nominal composition, and the melting temperatures remain largely consistent with that of CsNiCl_3_. Since neither a systematic change in the decomposition temperatures nor alterations in the melting temperatures are observed, we propose that the mixed samples are more likely to be a physical mixture rather than a chemical one, which is in agreement with predictions made by previous powder XRD analysis. Since hybrid perovskite solar cells are known to decompose below 100 °C, the high thermal stability of these nickel-based perovskites represents an advantage for their use in solar cells [30,31].

### 2.3. Raman Spectroscopy

**Figure 7:**
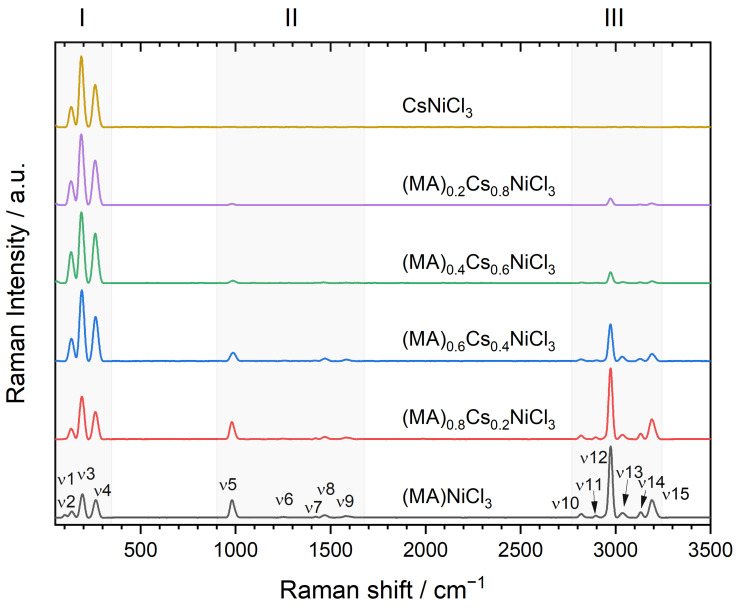
Raman spectra of (MA)_x_Cs_1−x_NiCl_3_ samples measured at room temperature. The ν1–ν15 modes are indicated in Table 2.

The Raman spectra recorded at room temperature for samples of mixed composition are shown in Figure 7. As is typical for hybrid perovskites, the Raman spectra can be analyzed in three spectral ranges, each with specific sources of vibrations [32]. In the low-energy range of 50–300 cm^−1^ (zone I), we observe the Metal-Halide and lattice modes; in the energy range of 900–1600 cm^−1^ (zone II), the CH3–NH3 modes are prominent; and in the range of 2800–3200 cm^−1^ (zone III), the individual N–H and C–H modes are present. The exact peak position for the observed vibrational modes was determined by fitting each curve and is summarized in Table 2.

The vibrational modes, denoted as ν1, ν2, ν3, and ν4, corresponding to the translational movements of MA^+^/Cs^+^ and the vibrations, Cl–Ni–Cl bending and Ni–Cl stretching, within the NiCl_6_ octahedron [33], undergo a continuous and systematic reduction in wavenumber as the Cs^+^ concentration increases. This same effect has been observed in hybrid perovskites of Pb when doping with the same ions, indicating that Cs^+^ is replacing CH_3_NH_3_^+^ in the structure, leading to structural contraction [34], which agrees with our finding by powder XRD analysis, in which discrete shifts of some diffraction peaks were detected and suggested that the replacement of MA^+^ by Cs^+^ occurs to a very small concentration. Raman signals detected from 900 cm^−1^ and above are characteristics of the methylammonium ion, as described previously. Of particular note is the mode labeled ν12, which remains constant for all samples containing methylammonium. This mode originates from the C-H stretching of CH_3_NH_3_^+^ [35]. Regarding the CsNiCl_3_ phase, the spectrum consisting of three signals in the measured range resembles very well the study reported by Jandl et al. [36] and is very similar to those reported in single crystals [37,38] and NiCl_2_ [39].

**Figure 8:**
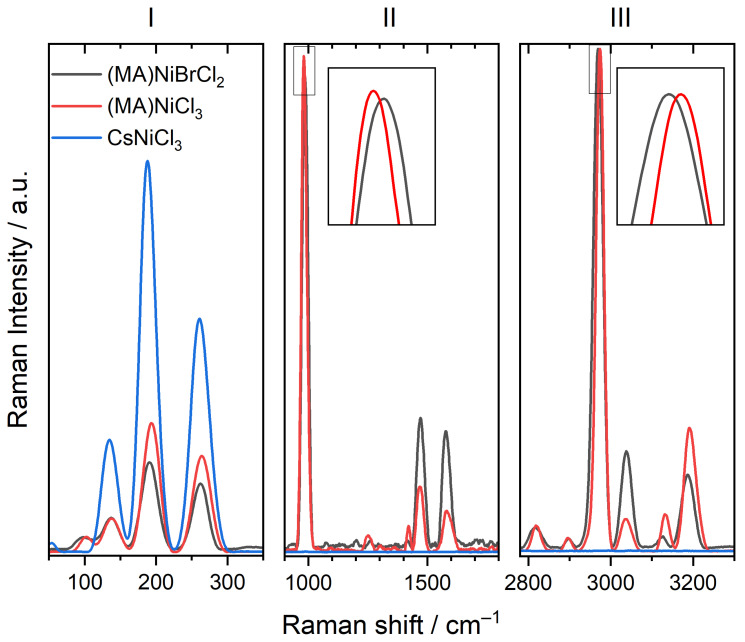
Raman spectra of samples (MA)NiCl_3_, (MA)NiBrCl_2_, and CsNiCl_3_, detailed in the three characteristic zones of hybrid perovskites.

The samples of composition (MA)NiCl_3_, (MA)NiBrCl_2_, and CsNiCl_3_ are compared in Figure 8. The incorporation of Br into the crystal lattice of (MA)NiCl_3_ introduces additional vibration modes associated with the Ni–Br bonds, which appear as new peaks in the spectra, superimposed on the existing Ni–Cl vibrations. Moreover, the presence of Br may influence the lattice dynamics within the crystal, leading to shifts in the frequencies of the existing modes. In general, it is expected that the heavier Br forms stronger bonds and, therefore, shifts the signal to lower frequencies. However, this shift depends on the specific vibration modes and details of the crystal structure, so it is possible to observe shifts to both higher and lower frequencies with the incorporation of Br. In Zone I of Figure 8, it is possible to distinguish the shift to lower frequencies for the sample (MA)NiBrCl_2_ compared to (MA)NiCl_3_. Zones II and III comprise vibration modes of the CH_3_NH_3_^+^ ion. As expected, in both zones, no major shifts are observed since the organic cation remains unchanged; however, small shifts are detected for ν5 (zone II) and ν12 (zone III), as shown in the insets of Figure 8. In the first case, the shift of the ν5 mode (C–N stretching) of (MA)NiBrCl_2_ to higher frequencies may be attributed to the loss of weak halogen–hydrogen interactions for bromine, allowing the C–N bond to stretch more freely [32,40]. On the other hand, the shift of the ν12 mode (CH_3_ asymmetric stretching) of (MA)NiBrCl_2_ to lower frequencies is because the introduction of Br in the lattice causes an increase in the unit cell volume, reducing the electrostatic interactions and therefore weakening the halogen-Ni bonds [41,42,43].

### 2.4. Optical Absorption

**Figure 9:**
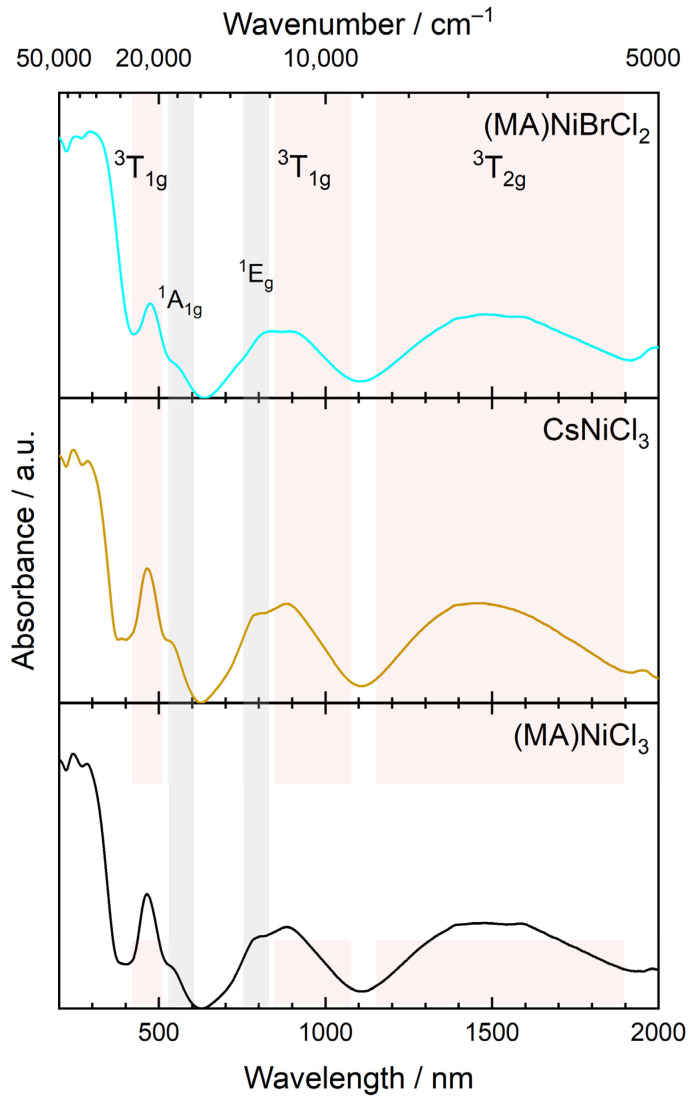
UV-VIS-NIR absorption spectra of samples (MA)NiCl_3_, CsNiCl_3_, and (MA)NiBrCl_2_.

The absorption properties of the samples were investigated by UV-V-NIR spectroscopy. It is well-known that Ni^2+^ compounds are optically active in the near-infrared and visible regions of the electromagnetic spectrum. The absorption spectra of all samples are typical for Ni^2+^ in an octahedral environment [44]. At room temperature, they consist of three broad absorption bands at approximately 1900–1200 nm, 1100–800 nm, and 500–400 nm, as shown in Figure 9. The electronic transitions responsible for these bands correspond to the spin-allowed transition from the ^3^A_2g_ ground state to ^3^T_2g_, ^3^T_1g_(F), and ^3^T_1g_(P) states, respectively. Additionally, spin-forbidden transition to singlet states ^1^E_g_ and ^1^A_1g_ are also distinguishable in the spectra. The broad spin-allowed bands arise from the spin-orbit splitting of the excited states [45], and the absorption maxima at energy higher than the ^3^T_1g_ state can be attributed to various spin-forbidden transitions [46]. The incorporation of Br^−^ into the (MA)NiCl_3_ lattice does not have a clear impact on the electronic absorption spectra. However, a careful examination of the values in Table 3 reveals a redshift. According to ligand field theory, the less electronegative Br^−^ exerts a weaker crystal field effect on Ni^2+^, leading to a decrease in the energy gap between the *t*_2*g*_ and *e_g_* levels. This effect has already been observed and measured in nickel halide compounds [46,47]. In addition, Br^−^ incorporation broadens the absorption bands, which in turn can be leveraged by solar cells to capture a wider range of wavelengths. Bandgaps were determined using the Tauc method based on diffuse reflectance measurements. For CH_3_NH_3_NiCl_3_ and CsNiCl_3_, the obtained values are 1.14 eV and 1.17 eV, respectively, while for the mixed samples, the bandgaps lie between these two values. We found reference data for CsNiCl_3_, calculated by DFT and GGA functional, to be 0.80 eV and predicted to be an indirect bandgap semiconductor [48,49]. It is well-known that GGA underestimates bandgap values [50]; therefore, our experimental results can be considered accurate.

## 3. Materials and Methods

### 3.1. Synthesis

Synthesis and sample manipulation were carried out under an argon atmosphere. All chemicals were used as received without further purification. Polycrystalline CH_3_NH_3_NiCl_3_, CsNiCl_3_, and mixed-composition samples were prepared by solvent evaporation from the stoichiometric mixture of the precursors. In a typical experiment, stoichiometric amounts of NiCl_2_ (Sigma Aldrich, 99%), CsCl (Sigma Aldrich, 99.9%), and CH_3_NH_3_Cl are dissolved together in 5 mL of ethanol (Sigma Aldrich, ≥99.9%). The green mixture was stirred for 4 h at room temperature and then evaporated at 50 °C until an orange powder precipitated. The product was washed with ethyl ether several times and vacuum dried at 60 °C for 24 h, then stored under an argon atmosphere.

CH_3_NH_3_Cl was prepared by the direct reaction of methylamine and hydrochloric acid. In short, a concentrated aqueous solution of hydrochloric acid (48 wt. %. Sigma Aldrich) was reacted with methylamine (40 wt. % in water. Sigma Aldrich) at 0 °C for 2 h with constant stirring. The mixture was then evaporated at 50 °C, and the resulting white precipitate was washed with ethyl ether three times and then vacuum dried at 60 °C for 24 h. 

### 3.2. Characterization

X-ray powder diffraction experiments were conducted at room temperature using a Bruker (Billerica, MA, USA) D8 Advance instrument, with copper Kα radiation (l = 1.5406 Å) in the range 5° ≤ 2q ≤ 70°. Full-profile refinements were carried out using the Rietveld method as implemented in the Jana 2020 software [51].

Raman spectra were acquired using a Confocal Raman Microscope, Jasco (Easton, MD, USA) NRS-4500 model, equipped with an air-cooled Peltier CCD detector and a 532 nm wavelength laser. The scanned range was from 50 to 4000 cm^−1^, with a data interval of 1 cm^−1^. Each spectrum was collected as an accumulation of 2 scans, each scan lasting 5 s. A laser power of 0.9 mW was used to prevent damage to the samples.

Diffuse reflectance measurements were performed using a UV-V-NIR spectrophotometer, Jasco (Easton, MD, USA) V-770 model, on polycrystalline samples at room temperature. The measurements were conducted in the range 200–2000 nm. with a scan speed of 400 nm min^−1^ and a data interval of 0.5 nm. The optical bandgap was estimated using the Tauc method.

The thermal behavior of the samples was investigated using thermal gravimetric analysis and differential scanning calorimetry (TGA-DSC) on a Netzsch (Selb/Bayern, Germany) Jupiter instrument. The heating and cooling rates were set to 10 K min^−1^, with a purge gas flow of N_2_ at 50 mL min^−1^. Measurements were conducted in the temperature range of 20 to 900 °C.

## 4. Conclusions

This study has successfully synthesized and characterized a series of nickel-containing hybrid perovskites, specifically (MA)NiCl_3_, CsNiCl_3_, and (MA)NiBrCl_2_, along with their structural variants through cation and halide substitution. X-ray powder diffraction analysis confirmed distinct crystal structures for (MA)NiCl_3_ and CsNiCl_3_, indicating the critical role of the cation in defining the crystallography of these materials. The substitution of Cs^+^ for MA^+^ and Br^−^ for Cl^−^ led to subtle but significant changes in the crystal structures, demonstrating the tunability of these perovskites through compositional adjustments. The thermal analysis provided evidence of the high thermal stability of the synthesized materials, with decomposition temperatures suitable for various applications. UV-VIS-NIR spectroscopy revealed characteristic absorption bands associated with Ni^2+^ ions, with shifts observed upon bromide substitution. These shifts suggest that the optical properties of these perovskites can be fine-tuned through halide modification. Raman spectroscopy further supported the structural characterization, providing insights into the vibrational modes of these compounds. The observed shifts in vibrational frequencies with substitution offer additional evidence of the structural changes.

Our findings underscore the significant potential of nickel-containing hybrid perovskites, particularly (MA)NiCl_3_, CsNiCl_3_, and (MA)NiBrCl_2_, for applications in energy conversion and storage technologies. The ability to tailor the structural, thermal, and optical properties of these materials through cation and halide substitution enhances their appeal for a wide range of applications. Importantly, the tunable properties highlighted in this study suggest these materials could play a crucial role in the development of efficient electrocatalysts for water splitting, a promising avenue for sustainable energy production. Future work will not only focus on optimizing these substitutions to develop materials with targeted properties for specific applications but will also extensively test these perovskites as electrocatalysts for water splitting. This direction aims to contribute significantly to the search for renewable energy solutions, further expanding the utility of these versatile compounds in the field of material science and energy technology.

## Figures and Tables

**Table 1:** Lattice parameters of samples (MA)_1−x_Cs_x_NiCl_3_, refined by the LeBail method.

Sample	(MA)NiCl_3_, Orthorombic *Cmcm*				CsNiCl_3_, Hexagonal *P6_3_/mmc*		
	*a*/Å	*b*/Å	c/Å	*V*/Å^3^	*a*/Å	*c*/Å	*V*/Å^3^
(MA)NiCl_3_	6.980 (2)	14.796 (6)	5.953 (2)	614.8 (4)	-	-	-
(MA)_0.8_Cs_0.2_NiCl_3_	6.978 (3)	14.774 (7)	5.950 (3)	613.5 (5)	7.183 (2)	5.938 (3)	265.3 (1)
(MA)_0.6_Cs_0.4_NiCl_3_	6.982 (6)	14.772 (9)	5.917 (7)	610.2 (9)	7.177 (2)	5.942 (3)	265.1 (2)
(MA)_0.4_Cs_0.6_NiCl_3_	6.983 (2)	14.760 (4)	5.925 (2)	610.7 (3)	7.178 (2)	5.940 (1)	265.0 (1)
(MA)_0.2_Cs_0.8_NiCl_3_	6.963 (2)	14.742 (5)	5.931 (2)	608.8 (3)	7.173 (2)	5.924 (2)	264.0 (1)
CsNiCl_3_	-	-	-	-	7.176 (1)	5.917 (2)	263.9 (1)

**Table 2:** Raman modes are derived from room temperature measurements. The vibrational modes discussed in the main text are in bold.

	(MA)NiCl_3_	(MA)_0.8_Cs_0.2_	(MA)_0.6_Cs_0.4_	(MA)_0.4_Cs_0.6_	(MA)_0.2_Cs_0.8_	CsNiCl_3_
ν1	102.08	100.08	90.54	-	-	-
ν2	138.08	135.08	136.54	134.54	133.54	132.08
ν3	194.08	191.08	190.54	188.54	187.54	186.08
ν4	264.08	262.08	262.54	261.54	260.54	258.08
ν5	981.08	980.08	987.54	986.54	979.54	-
ν6	1252.08	1248.08	1256.54	-	-	-
ν7	1421.08	1423.08	1418.54	1425.54	1422.54	-
ν8	1468.08	1470.08	1468.54	1464.54	-	-
ν9	1582.08	ν1582.08	1583.54	1579.54	-	-
ν10	2819.08	2818.08	2817.54	2823.54	2812.54	-
ν11	2896.08	2894.08	2901.54	2898.54	2891.54	-
ν12	2974.08	2973.08	2973.54	2973.54	2973.54	-
ν13	3035.08	3036.08	3034.54	3036.54	3042.54	-
ν14	3132.08	3132.08	3129.54	3130.54	3130.54	-
ν15	3191.08	3191.08	3191.54	3189.54	3190.54	-

**Table 3:** Transition wavelength, in nm, from the ground state ^3^A_2g_ of Ni^2+^ in the samples (MA)NiCl_3_, (MA)NiBrCl_2_, and CsNiCl_3_.

	CH_3_NH_3_NiCl_3_	CH_3_NH_3_NiBrCl_2_	CsNiCl_3_
^3^T_2g_	1493	1482	1453
^3^T_1g_	885	905	884
^1^E_g_	793	822	793
^1^A_1g_	541	555	537
^3^T_2g_	464	476	465

## Data Availability

Data is available on request from the authors.
